# Serology for *Borrelia* spp. in Northwest Italy: A Climate-Matched 10-Year Trend

**DOI:** 10.3390/life11121310

**Published:** 2021-11-27

**Authors:** Giacomo Stroffolini, Francesco Vladimiro Segala, Tommaso Lupia, Silvia Faraoni, Luca Rossi, Laura Tomassone, Stefania Zanet, Francesco Giuseppe De Rosa, Giovanni Di Perri, Andrea Calcagno

**Affiliations:** 1 Infectious Diseases Unit, Department of Medical Sciences, Amedeo di Savoia Hospital, University of Turin, 10149 Turin, Italy; giovanni.diperri@unito.it (G.D.P.); andrea.calcagno@unito.it (A.C.); 2 Clinic of Infectious Diseases, Catholic University of the Sacred Heart, 00168 Rome, Italy; 3 Unit of Infectious Diseases, Cardinal Massaia Hospital, 14100 Asti, Italy; tommaso.lupia89@gmail.com (T.L.); francescogiuseppe.derosa@unito.it (F.G.D.R.); 4 Laboratory of Microbiology and Virology, Ospedale Amedeo di Savoia, ASL “Città di Torino”, 10149 Turin, Italy; silvia.faraoni@aslcittaditorino.it; 5 Department of Veterinary Sciences, University of Turin, 10149 Turin, Italy; luca.rossi@unito.it (L.R.); laura.tomassone@unito.it (L.T.); stefania.zanet@unito.it (S.Z.)

**Keywords:** tick-borne-diseases, climate change, *Borrelia* spp., northwest Italy, zoonosis, ticks

## Abstract

Ticks are hematophagous parasites that can transmit a variety of human pathogens, and their life cycle is dependent on several climatic factors for development and survival. We conducted a study in Piedmont and Aosta Valley, Italy, between 2009 and 2018. The study matched human sample serologies for *Borrelia* spp. with publicly available climatic and meteorological data. A total of 12,928 serological immunofluorescence assays (IFA) and Western blot (WB) tests were analysed. The median number of IFA and WB tests per year was 1236 (range 700–1997), with the highest demand in autumn 2018 (*N* = 289). In the study period, positive WB showed an increasing trend, peaking in 2018 for both IgM (*N* = 97) and IgG (*N* = 61). These results were consistent with a regional climatic variation trending towards an increase in both temperature and humidity. Our results suggest that coupling data from epidemiology and the environment, and the use of a “one health” approach, may provide a powerful tool in understanding disease transmission and strengthen collaboration between specialists in the era of climate instability.

## 1. Introduction

Climate change refers to a long-term change in average weather patterns [1] with a broad range of effects on global health [2] and, according to the latest report by The Intergovernmental Panel on Climate Change (IPCC), this shift is “unequivocally influenced” by human activities [3]. In terms of infectious diseases, increasing land and ocean temperatures, along with changes in precipitation patterns and extreme weather events, are all factors that may promote the geographic expansion of communicable diseases [4]. In this context, vector-borne diseases are particularly sensitive to climate variations, as even small changes in weather patterns may significantly increase climate suitability to arthropods, thus leading to a global emergence, resurgence, and redistribution of vector-borne infectious diseases [5].

Tick-borne diseases (TBDs) are a group of heterogenic clinical entities with complex transmission cycles conditioned by tick phenology, vertebrate hosts’ biology, and habitat characteristics. In Europe, the main tick vector able to transmit pathogens with endemic potential is *Ixodes ricinus*, which lives in humid environments and typically thrives in deciduous woodlands and mixed forests. In fact, *Ixodes* spp. is the vector of *Borrelia burgdorferi* sensu lato (s.l.), the agent of Lyme borreliosis, which is the most prevalent tick-borne infection in the northern hemisphere. Different *Borrelia burgdorferi* genospecies with pathogenic potential are present in Europe, generally causing skin rash (*erythema migrans*) and non-specific clinical manifestations, mainly affecting the nervous system and joints [6]. *Ixodes ricinus* also transmit *B. miyamotoi* (causing tick-borne relapsing fever) and other *Borrelia* spp., *Anaplasma phagocytophilum* (human granulocytic anaplasmosis (HGA)), tick-borne encephalitis virus (TBEV), *Ehrlichia* spp. (ehrlichiosis), *Babesia* spp. (babesiosis), *Rickettsia* spp. (rickettsiosis), *Francisella tularensis* (tularemia) and *Bartonella henselae* (bartonellosis) [7,8]. Other tick species involved in disease transmission in Europe are *Rhipicephalus sanguineus*, vector of *Rickettsia conorii* (Mediterranean Spotted Fever) [9], and *Dermacentor* spp., vector of TBEV, Omsk hemorrhagic fever viruses, *Rickettsia* spp. and *F. tularensis*. In addition, *Hyalomma* spp. has recently been reported to be responsible for many Crimean-Congo hemorrhagic fever cases in Eastern and Southwestern Europe [10,11,12].

Ticks are obligate hematophagous ectoparasites of animals and humans and, as is the case for many other arthropods, their life cycle is dependent on several climate factors for development and survival. Thus, changes in their distribution range can be influenced by climatic, environmental and anthropogenic factors. Regarding climate, warmer temperatures have been shown to have positive effects on the phenology of exophilic tick vectors, such as *I.* *ricinus*, especially at the limits of their latitudinal and altitudinal range [13]. Higher temperatures favour oviposition, egg development and interstadial development of *I. ricinus* [13,14,15]. Moreover, extended vegetation periods due to shorter winters increase the time ticks spend questing in the environment [14], and warmer temperatures can promote tick dispersion and establishment in new geographic areas. For example, *Hyalomma* ticks are increasingly reported at northern latitudes, where they are passively transported through migrating birds [16]. This can lead to the spread of pathogens in new geographic areas, where they can also adapt to new local vectors.

Good-quality surveillance on the spatio-temporal trends of tick-borne diseases is generally lacking, but it is reasonable to believe that their incidence is increasing in several regions due to better climate suitability. The biological and eco-climatic requirements for Ixodes’ survival are vertebrate host availability (i.e., rats, birds, lizards and ungulates), temperatures included within −10 and +35 °C and relative humidity higher than 80% [16,17], all factors that are widely present in Europe, including northern Italy. In fact, in this geographical area, despite the lack of a systematic surveillance system, the number of cases of tick-borne encephalitis and Lyme borreliosis are showing an increasing trend [17,18,19,20]. This tendency also appears to be reinforced, for both *Hyalomma* and *Ixodes* spp., by predictive models taking into account geostatistics and different climate scenarios [21,22]. This is under debate not only for classical involved areas but across ecosystems that may vary significantly in the coming years [1,2,3,4,22].

The purpose of the study was to assess the epidemiological variables and characteristics of *Borrellia* spp., its distribution and its role at the human–environmental interface in the Piemonte and Aosta Valley area. These two regions are located in northwestern Italy, are surrounded by the Alps and border France and Switzerland. The geographical configuration of the regions mainly consists of mountains, with an area of flat plains and hills in the southern part of Piedmont, where the urban and peri-urban area develops. A population of around 4 million inhabitants is registered in Piedmont (surface: 25,386.7 Km^2^), while around 124,000 inhabitants live in Aosta Valley (surface: 3260.8 Km^2^) [23]. For these features, we aimed to describe the changing picture in the area, as the regions may be a good field in which to assess the evolving epidemiological characteristics of *Borrelia* spp. infections, also for being a site of major human–environmental interactions, and for being located at an international cross-border crucial area.

## 2. Materials and Methods

We retrospectively included data from our hub regional reference laboratory, Ospedale Amedeo di Savoia, Turin, Italy; testing was performed at our institution for clinical reasons during 2009–2018. Our institution is the hub regional reference laboratory, processing samples across the region and from bordering areas without means for validating tests and that have low testing capacity.

Plasma samples were collected between 2009 and 2018 and subjected to serology or Western-blot (WB) for suspected TBD-related infections. Clinical, demographic and laboratory characteristics were recorded at the time of presentation. All of them were labeled with additional data, specifying geographical area/laboratory of provenience. Serology and WB for *Borrelia* spp. were performed by chemoluminescence technique (“LIAISON Borrelia IgM and IgG–DiaSorin”) and immunoblot “recomLine Borrelia Ig Mikrogen Diagnostik” (IgG/IgM kit); both are validated tests and are able to identify *Borrelia burgdorferi* sensu stricto, *B. garinii*, *B. afzelii*, *B. bavariensis*, *B. spielmanii*, as per manifacturer indication, with high sensitivity and specificity. The retrospective analysis of the collected data was approved by the Ethics Committee “Malattie da Vettore, ASLCITTADITORINO”.

We matched data from our analysis with consulted regional publicly available data for climate variables from ARPA: historical meteorology database of the Regional Agency for Environment Protection (ARPA Piemonte, https://www.arpa.piemonte.it, accessed on 12 October 2021) [24].

Since Lyme borreliosis is a notifiable disease in Italy, we also matched our data with notifications from the Piedmont Regional Service for the Epidemiology of Infectious Diseases (SEREMI) (available at https://www.seremi.it/pubblicazioni, accessed on 12 October 2021). We performed descriptive statistics on the entire study population and then stratified between study participants according to geographical or laboratory of provenience, seasonality and test performed. Data were analysed using standard statistical methods. Variables were described with medians, absolute values and rates. Data analysis was performed using SPSS software for Mac (version 27.0. IBM Corp, Armonk, New York City, NY, USA). Graphs were created with SPSS and ppt.

## 3. Results

**Figure 1:**
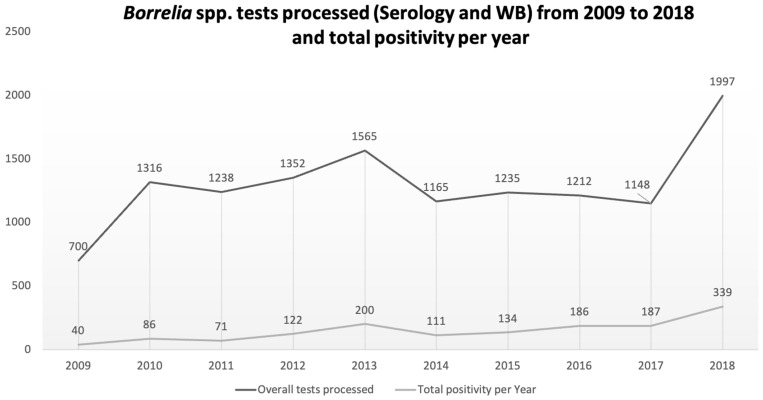
Tests requested for *Borrelia* spp. per year showing the overall trend and total number of positive tests per year.

A total of 12,928 serological immunofluorescence assays (IFA) and western blot (WB) tests for *Borrelia* spp., performed at the Microbiology Laboratory of the Amedeo di Savoia Hospital over a period of 10 years, from 1 January 2009 to 31 December 2018, were included in the final analysis (Figure 1). Samples lacking precise test date were excluded from the analysis.

**Figure 2:**
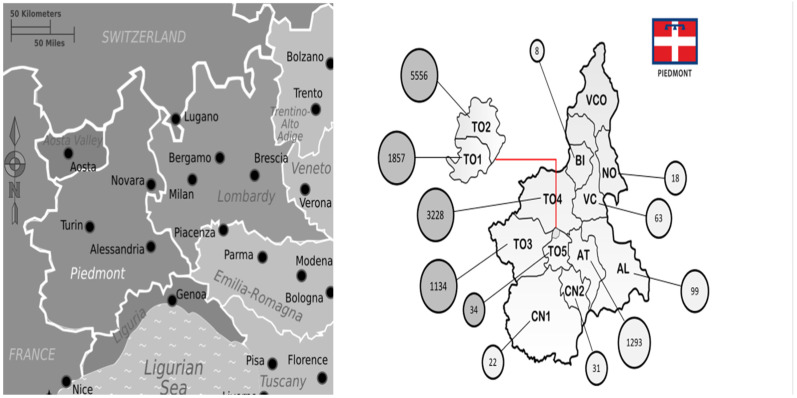
Map of the Piedmont region (**on the right**) and its geographical location (**on the left**); the region is subdivided in areas of interest. Each circle represents the relative magnitude of the number of tests coming from the area. The Turin (Torino) territory, as all Italy, is subdivided in different “ASL” (sanitary districts) that pertains to an area of interest, with different numberings; AL: Alessandria; AO: Aosta AT: Asti; CN: Cuneo; VC: Vercelli; NO: Novara; BI: Biella; VCO: Verbania-Cusio-Ossola.

All tests referred to Piedmont and Aosta Valley residents. The highest number of tests were requested in 2018 (*N* = 1997) and the lowest in 2009 (700). The median number of IFA and WB tests per year in the period 2009–2018 was 1236 (range 700–1997), with a peak in 2018 (*N* = 1997). People involved in testing were male in the 59.3% of cases with a median age of 47 (18–78). The tests came from different parts of the Piedmont region (13.327; 97.3%) as shown in the Figure 2 and from Aosta Valley region (99; 2.7%).

**Figure 3:**
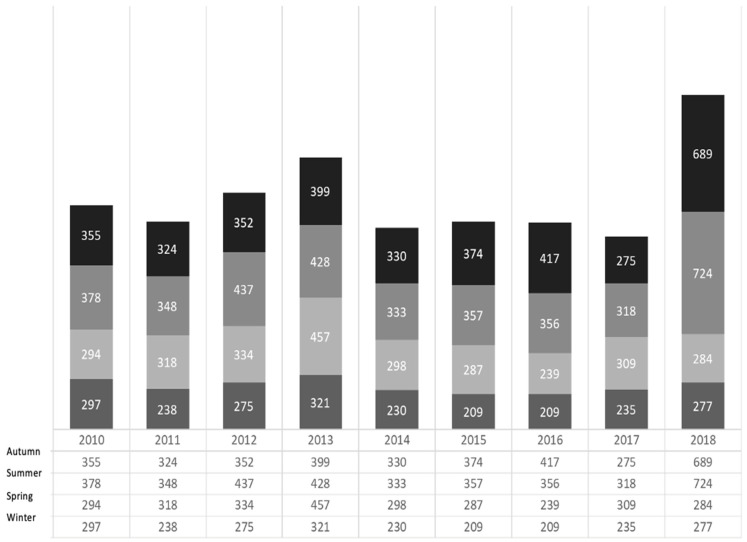
Trend in testing between 2010 and 2018, as per seasonal number of tests.

The total sample of IFA and WB tests (excluding those from 2009, which were excluded from the analysis due to the lack of a precise testing date) was stratified by season into four groups: autumn (from September to November), winter (from December to February), spring (from March to May) and summer (from June to August) (Figure 3).

The median number of tests (both IFA and WB) analysed during autumn season was 355 (range 275–689), with the highest test demand occurring in autumn 2018 (*N* = 689) and the lowest in 2011 (Figure 3). In winter, the median number of tests analysed in our laboratory was 238 (range 209–321), reaching a peak in winter 2013 (*N* = 321) and the lowest numbers in both 2015 and 2016 (*N* = 209). The median number of tests analysed in spring was 355 (range 284–457), with the highest test demand occurring in spring 2013 (*N* = 457), the lowest in 2016 (*N* = 239). Finally, in summer, the median number of tests was 238 (range 318–724), peaking in 2018 (*N* = 724), with the lowest in 2017 (*N* = 318).

**Figure 4:**
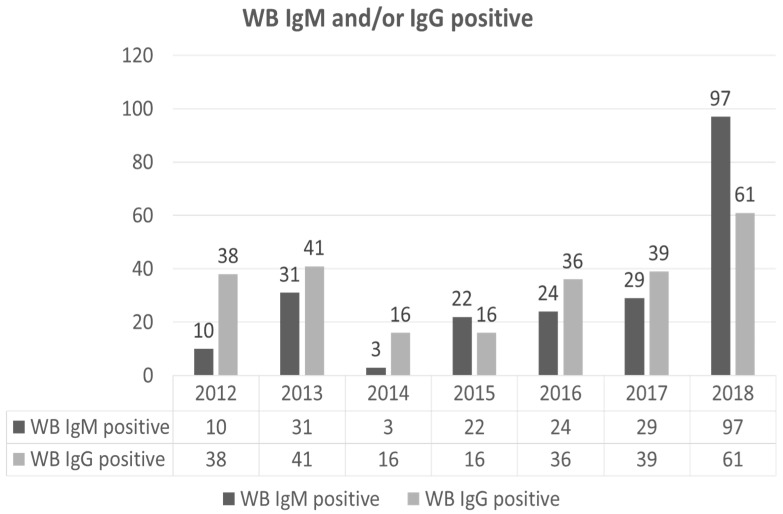
Number of WB testing positive over the years, IgM and IgG.

We also stratified the data on WB tests according to test type (years 2009–2011 were excluded from the analysis due to a lack of data) (Figure 4).

Positive WB IgM tests ranged from 3 to 97 per year in the period of analysis (median 24), with a peak in 2018 (*N* = 97). Positive WB IgG tests ranged from 16 to 61 per year in the period of analysis (median 38), peaking in 2018 (*N* = 61).

We matched our data with SEREMI notification system (Table 1).

## 4. Discussion

### 4.1. Epidemiology of TBDs in Europe, Italy and Piedmont

Globally, the incidence/prevalence of tick-borne diseases is rising, mostly due to increased interactions between pathogens, vectors and hosts. Some of the most important factors that account for the increasing incidence include urbanization and human population growth, behavioural changes such as human encroachment into natural environments, climate and habitat changes, and increased wildlife populations in urban and peri-urban areas [26,27].

Lyme borreliosis is widespread in Europe [28] and anti-B. burgdorferi antibodies are present in the human population with a prevalence that varies considerably between geographical areas (from 0% to 23.2%) [28,29]. This is of high importance in view of the climate and habitat changes not only intervening largely in Europe but specifically across the Alps, an environment of great interest with peculiar aspects that act as frontier and a way of communication at the same time [30].

In the Alpine region, case notifications of Lyme borreliosis have been increasing in recent years, both in Italy [20] and in Switzerland [31]. This increase is accompanied by the geographical expansion of ticks in the western Alps [29]. Various field surveys have been carried out broadly in Italy and specifically in Piedmont ad Aosta Valley regions [32,33,34,35,36,37,38,39,40,41,42,43], where questing ticks and ticks collected from animal hosts harboured several *Borrelia* spp., mainly *B. afzelii* and *B. garinii* and other tick-borne pathogens, such as *Babesia* and *Rickettisa* spp. [29,32,35,37,38,40,41,43].

Studies [34,36,37] analysing ticks (mostly *Ixodes* spp.) found on patients showed up to 22.8% of pathogens’ positivity in risk population in the region, with a prevalence of *Borrelia* spp. of about 6%.

### 4.2. Our Data in the Picture Frame

Our regional reference centre for infectious diseases is a hub for clinical and laboratory testing. However, there is no uniformity in the clinical approaches taken by our institutions because different clinical units take different approaches towards TBDs and no dedicated outpatient service is available. Nonetheless, we can assume that the increasing number of tests for *Borrelia* spp. over the years reflects an increasing human risk of exposure to TBDs and, in general, could be considered a good proxy for host–vector contact [16,17,30]. Unfortunately, due to the nature of the samples and the COVID-19 pandemic, we could not find any additional information about the primary cases. The majority of the samples at our institution come from the western parts of the region (ASLTO3 and ASLTO4) and Turin city. The former are documented, important human/vector/wildlife interface areas [32,33,34,35,36,37,38], but when it comes to the Turin area, it is reasonable to assume that the majority were ‘referral’ samples, meaning that the vast majority of these came from people who had accessed surrounding areas during recreational outdoor activities, experienced some kind of exposure to ticks and were subsequently seeking medical attention for symptomatic/asymptomatic exposure [40,42].

It is worth noting that, overall, the number of test requests has continuously grown over the years. As already proposed, this is a good proxy for human–vector–host contact and our data may show that this has constantly been increasing. However, other factors could be proposed as explanations for this, including increasing clinician awareness, availability of testing, confidence in exam interpretation and population knowledge about the link between ticks and diseases. Moreover, the role of serology in surveillance is debated, displaying inherent limitations, but we pursued an approach based on literature data and performed reliable validated serology. Unfortunately, the time-span persistence of these Ig, in absence of follow-up, cannot be assessed, and we know from literature that measuring the antibodies’ trajectory may be unreliable [6,44,45,46]. Testing nearly doubled between 2009 and 2010. It then remained quite stable over some years, except for a blip in 2013, before it started rising again in 2018. Not surprisingly, summer was the best represented season for most of the surveyed years. In late spring and summer, the abundance of *I. ricinus* nymphs peaks [13,14,16], and in parallel, humans (e.g., hikers, mountain bikers and mushroom pickers) undertake more outdoor activity in forested and humid areas favourable for tick reproduction and survival, thus favouring contact between the vector and the pathogen. Interestingly, in 2013, a year in which the median temperature in spring stayed lower for longer than usual for all provinces except Novara, from where only the minority of our data came from, there were more reported cases in spring than summer. However, despite the lower average temperature, humidity increased. In contrast, in 2015 and 2016 there were more reported cases in autumn than spring and summer, being respectively the eleventh and ninth hottest autumn seasons ever registered in the region, particularly across the western Alps and eastern areas during September. Again, for these years, these periods were humid [24]. Much of the data indicate that the relationship between TBDs and temperature is non-linear and not unimodal, and, as already pointed out, humidity plays an important role. Matching these variables may indicate a general trend of an increasing number of hosts and vectors and favourable conditions for their maintenance. The majority of our data came from the northwestern and metropolitan quadrants of the region, the most studied for the presence of ticks [32,33,34,35,36,37,38,39,40,41,42,43]. This is no surprise if we consider the habitat and the good collaboration between medical and veterinary centers in the area. More data from other parts of the region on environmental surveillance might improve these observations.

An additional finding regarding the winter season deserves attention. Generally speaking, we observed an unexpectedly high number of test requests during winter. From one side, the tick’s niche has evolved over time, making possible the contact between human and vectors during unexpected periods and in unusual altitude, vegetation and climate features [29]. On the other side, we consider it sensible to think that there may have been a time lapse between when the patients became aware of any presenting symptoms and when they came to primary clinical attention. This is also because TBDs are commonly overlooked, misdiagnosed or tardively considered a possible explanation for a variety of symptoms, even in endemic regions [6,44,45,46]. Another possible explanation for this delay is the difficulty in accessing care in our medical system, especially between primary and referral centers. These factors may lengthen the period between when the actual contact between the vector and human occurred and when the test arrived at our laboratory. This is also in line with the high number of tests received during autumn. All these seasonal data are consistent with previous observations [29].

Looking closer at the data, a high number of WBs resulted in positives. We could not assess the seasonality of the WB analysis because of the low number of samples and their heterogeneity. The majority of these infections, if confirmed, were not constantly reported when we compared these data to those coming from the SEREMI system, which in turn depends on regional reporting systems. The delta reporting was surprising: 39 cases of Lyme disease were considered by SEREMI between 2016 and 2018 for the whole Piedmont region, the majority of which were referred between 2016 and 2018 (12 and 21, respectively) and mainly originated from the northernmost province (VCO) that relies on an independent testing centre [41]. In 2010, SEREMI reported three Lyme disease cases, and in subsequent years, the figure did not change remarkably (one case in 2012, three in 2013, four in 2014, five in 2015, and none in the other years of interest). Accordingly, the mismatch between the official caseload and the data collected at our reference institution was obvious (Table 1). The underreporting and misreporting of TBDs is of concern in clinical and epidemiological medicine [6,16,44,45,46], suggesting that awareness-raising policies amongst public and private medical actors should be prioritized with a view to strengthening the reliability of passive surveillance data. In this survey, contact with tick-borne *Borrelia* spp. agents apparently rose during the investigated 7-year time interval, reaching a maximum number in 2018 (97 IgM, 61 IgG positive). While a few false-positive results may not be excluded and IgG may simply confirm a past infection, IgM data warn about the actual risk of exposure to *Borrelia* spp. infection and, possibly, other tick-borne pathogens in the study area [6,44,45,46]. Furthermore, epidemiological and entomological surveys previously carried out in the same study area [32] identified 15.51% of the Piedmont regional territory as suitable for *Babesia* spp. presence and confirmed how areas with higher abundance of *I. ricinus* also have higher TBP prevalence values.

### 4.3. The Impact of Climate Change and Human Behaviour on the Incidence of TBD

The increase in the reported incidence of Lyme disease in Europe could be the result of a variety of factors, including an increase in the abundance of the vector, more abundant wildlife tick hosts, greater awareness and better diagnostics, and increased exposure of people [22]. Therefore, it is not straightforward to attribute changes in the distribution and abundance of ticks and tick-borne diseases to climate changes, as changes in vertebrate host populations, habitat and human exposure to ticks can all be related to the climate [26]. Despite that, more favourable conditions for tick propagation have been highlighted in this and other works [4,8,9,13,16,22,31]. The general trend in alps temperature [30], an area where many hosts graze, has been shown to be a relevant factor in other TBDs and it may be relevant in the future for *Borrelia* spp. [8,9,17,18,19,29]. The human–environmental interface has broken and ticks are now found in urban and peri-urban areas, colonizing districts historically out of their reach and pathogen-rich ticks are increasingly found on privately-owned dogs [35]. Data from Europe and our area indicates an important prevalence of pathogens also in ticks removed from humans that appeared to be more relevant for *Babesia* spp., but improved testing in larger samples may lead to *Borrelia* spp. identification, as it is largely present in the region. Land use changes, such as the transformation of natural ecosystems into residential or recreational areas, and the restoration of natural areas connectivity for biodiversity purposes, facilitate human contact with these vectors. It appears urgent to improve educational programs on personal protection and surveillance on TBDs in order to face the threat posed by tick vectors in green urban landscapes [40].

These data from the environment, human, vectors and animals highlight the continuous ongoing sylvatic, rural and urban cycles, but little is known about the human resulting burden of disease. To date, no large relevant studies did test for human serology samples in our area. Many TBDs are also difficult to diagnose at a laboratory level, because of the overall low sensitivity of available techniques. This is debatable for *Borrelia* spp. testing, for which laboratory techniques are able to identify evidence of exposure with higher sensitivity and specificity.

In summary, studies point toward the fact that TBDs are not limited or confined to sylvatic and rural environments but are increasingly reported in anthropic biological communities (human, pet and ectoparasites). In northwestern Italy (and specifically in the Piedmont region), the roe deer population is experiencing a steady increase and territorial expansion [39] that have driven a concomitant increase in ticks, especially *Ixodes ricinus*, which is the most common tick of temperate regions. Together, with ungulates, the number of ticks has also increased and their geographical range has expanded to include areas at higher latitude and/or altitudes, possibly due to the influence of climate changes on tick habitats [28,30]. Ultimately, questionnaire data shows how people infrequently adopt preventive measures, such as inspecting the body for ticks, as the risk perception remains low [42]. The limitations of our study include its retrospective nature, the lack of uniformity in testing criteria, and the lack of coupled clinical assessment. Moreover, we could not couple the serology results from WBs with PCRs or more accurate methods from human samples with a larger environmental surveillance database, except for some areas. Despite these limitations, our work shows some strength: It is by far the widest time interval in which such data have been registered in northwestern Italy, and it collected the highest number of samples for TBDs, especially *Borrelia* spp., in the region. Furthermore, it is the first study to attempt a coupling at the human-environment interface in the area. Ultimately, our serology testing is consistent with species found in the area, thus making these data more trustworthy. Additionally, we were able to highlight some interesting differences in seasonality, the presence of the bacteria, host and vector as well as the mismatch between reporting and the prevalence of *Borrelia* spp.

## 5. Conclusions

Deeper environmental and animal sampling is advised to complete the puzzle of an entangled and growing public health issue. In time, we will have to face big challenges due to climate variability. In view of this, reinforcing territory-based knowledge and coupling data from all fields, the use of a “one health” approach may provide a powerful tool to counteract and strengthen collaboration between specialists in the era of climate instability.

## Figures and Tables

**Table 1:** Mismatch for confirmed Lyme diseases cases between SEREMI surveillance system and the current report Ref. [25].

	2016	2017	2018
*Borrelia* spp. WB, IgM and IgG both positive (Current report)	36	39	61
SEREMI surveillance confirmed Lyme diseases	12	6	21

## Data Availability

The data presented in this study are available on request from the corresponding author.
